# Perihepatic caudate lobe haematoma originating from a pancreatic pseudoaneurysm: a diagnostic dilemma

**DOI:** 10.1093/bjrcr/uaae018

**Published:** 2024-06-07

**Authors:** Ippei Ozaki, Yohsuke Suyama, Kohei Hamamoto, Eiko Hyoe, Mai Fujisaku, Hiroshi Shinmoto

**Affiliations:** Department of Radiology, National Defense Medical College, Tokorozawa 359-8513, Japan; Department of Radiology, National Defense Medical College, Tokorozawa 359-8513, Japan; Department of Radiology, Jichi Medical University, Shimotsuke-shi, Japan; Department of Radiology, National Defense Medical College, Tokorozawa 359-8513, Japan; Department of Radiology, National Defense Medical College, Tokorozawa 359-8513, Japan; Department of Radiology, National Defense Medical College, Tokorozawa 359-8513, Japan

**Keywords:** perihepatic caudate lobe haematoma, lesser sac haematoma, ruptured hepatocellular carcinoma, pancreatic pseudoaneurysm, pseudoaneurysm in the pancreatic cyst, transarterial embolization

## Abstract

Despite advances in diagnostic imaging and interventional techniques, pancreatic pseudoaneurysms remain a life-threatening complication of pancreatitis. Presentation varies among patients and may include intra-abdominal, retroperitoneal, or gastrointestinal bleeding and bleeding into the pancreatic or common bile duct. We present a unique case of a 74-year-old man with a history of heavy alcohol consumption who presented with a haematoma surrounding the caudate lobe of the liver. Initially, alcoholic cirrhosis and a ruptured hepatocellular carcinoma were suspected. Therefore, transarterial embolization (TAE) of the caudate branch of the hepatic artery was performed. However, 3 months later, the patient experienced abdominal pain with a lesser sac haematoma and a seemingly interconnected pancreatic cyst. One month later, a pseudoaneurysm appeared in the pancreatic cyst. TAE was successfully performed for the pseudoaneurysm, and the patient showed no signs of recurrence during the 1-year follow-up.

## Introduction

Pancreatic pseudoaneurysms are a severe complication of pancreatitis and pancreatectomy. Although they generally occur within 3-5 weeks following pancreatitis,^1^ the timeline varies among cases, and the underlying mechanism remains poorly understood. Pancreatic pseudoaneurysms cause haemorrhage at various sites, such as the abdominal cavity, retroperitoneal cavity, gastrointestinal tract, pancreatic duct, or common bile duct.^2^ Herein, we report on a unique case of a patient who presented with a perihepatic caudate lobe haematoma of an unknown origin, which was eventually diagnosed as a pancreatic pseudoaneurysm 4 months later.

## Case presentation

**Figure 1.**
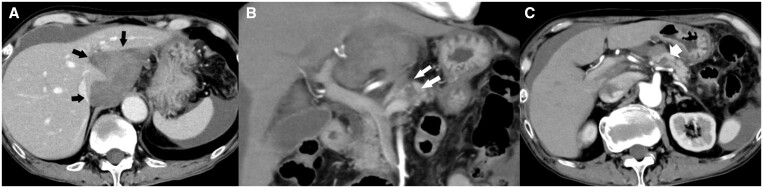
CT images taken before admission to our hospital. (A) Delayed-phase, contrast-enhanced, axial CT scan shows the haematoma around the caudate lobe of the liver (black arrow). (B) Delayed-phase, contrast-enhanced, coronal CT scan shows a small cyst in the pancreatic body (white arrow), which is connected to the haematoma. A tiny, hyperdense dot is seen at the upper pole of the cyst. (C) Arterial-phase, contrast-enhanced, axial CT also shows a small cyst in the pancreatic body (wide arrow). The hyperdense dot observed in the coronal image is not apparent here.

**Figure 2.**
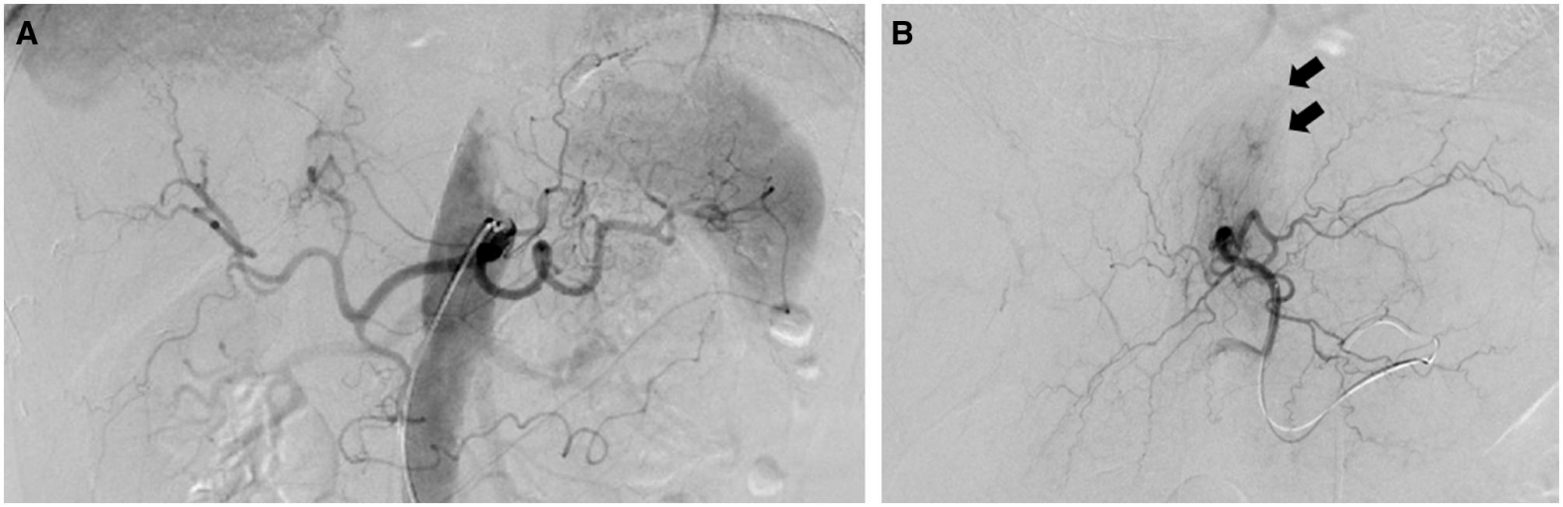
Angiography at the first admission. (A) Digital subtraction angiography of the celiac trunk shows no site of extravasation or pseudoaneurysm. (B) Digital subtraction angiography of the left hepatic artery shows abnormal enhancement of the hepatic caudate lobe (arrow).

A 74-year-old man, with a history of heavy alcohol consumption, was admitted to a local hospital with sudden abdominal pain. Dynamic CT revealed a haematoma surrounding the caudal lobe of the liver with an undetectable site of extravasation (Figure 1A). Considering the patient’s history of heavy alcohol consumption, alcoholic cirrhosis and a ruptured hepatocellular carcinoma (HCC) were initially suspected, and the patient was referred to our hospital for transarterial embolization (TAE). Blood tests revealed a lowered haemoglobin level (7.7 g/dL; normal range: 13.7-16.8 g/dL) and elevated serum pancreatic enzyme levels (amylase: 414 U/L [29-132 U/L], lipase: 411 U/L [7-66 U/L]). At 2.4 ng/mL, the level of alpha fetoprotein (tumour marker for HCC) was within the normal range (<10.0 ng/mL). A tiny, nonspecific cystic lesion, which seemed to be connected to the haematoma, was observed in the pancreatic body; a tiny hyperdense dot was observed within the cyst (Figure 1B and C). However, it was difficult to establish the cyst as the source of the haemorrhage because the tiny hyperdense dot appeared very small against the haematoma, and a connection with an artery was not observed. Furthermore, it was difficult to diagnose the cyst as a pancreatic pseudocyst because CT did not reveal other manifestations suggestive of acute or chronic pancreatitis, such as parenchymal enlargement or necrosis, fluid collection around the pancreas, pancreatic calculi, and ductal irregularity.^3^ Angiography, performed to detect the bleeding site, revealed no site of extravasation or pseudoaneurysms. However, an abnormal enhancement of the caudate lobe was observed (Figure 2). Considering that the haematoma was confined around the caudate lobe and an HCC rupture was suspected before admission to our hospital, we thought it might be possible that the caudate branch of the hepatic artery was responsible for the haematoma. Thus, the artery was embolized using a gelatine sponge, and the patient was discharged.

**Figure 3.**
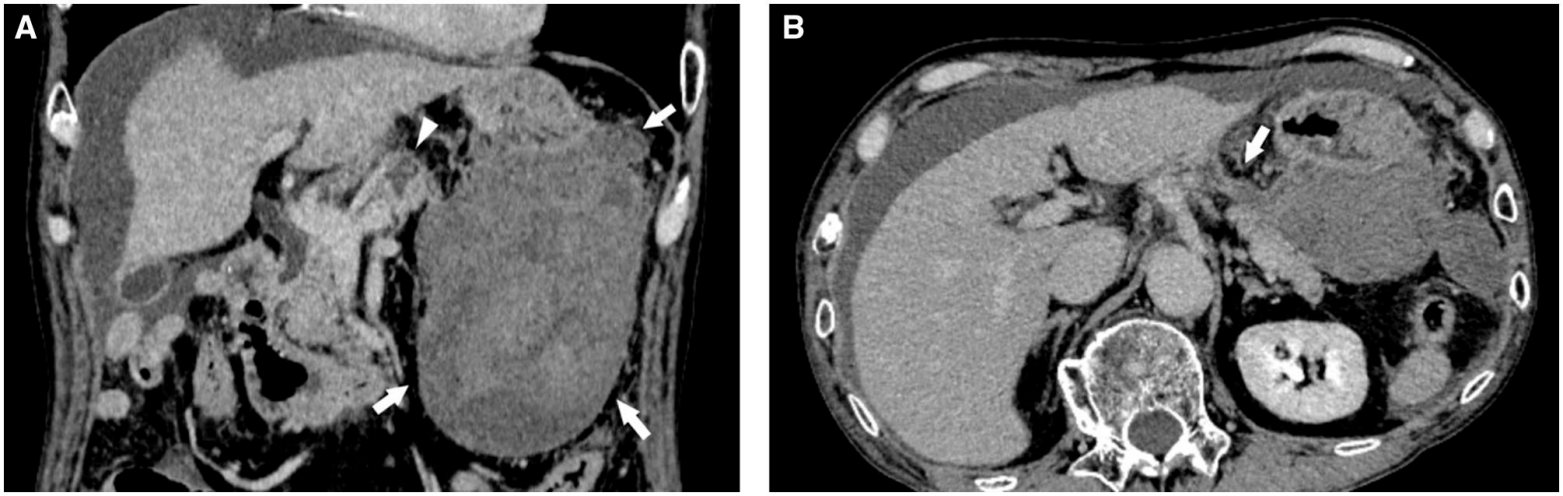
CT images obtained at the second emergency admission. (A) Coronal CT image shows a haematoma in the lesser sac (arrow) and a cyst in the pancreatic body (arrowhead). (B) Axial CT image suggests an interconnection between the haematoma and the cyst (arrow).

**Figure 4.**
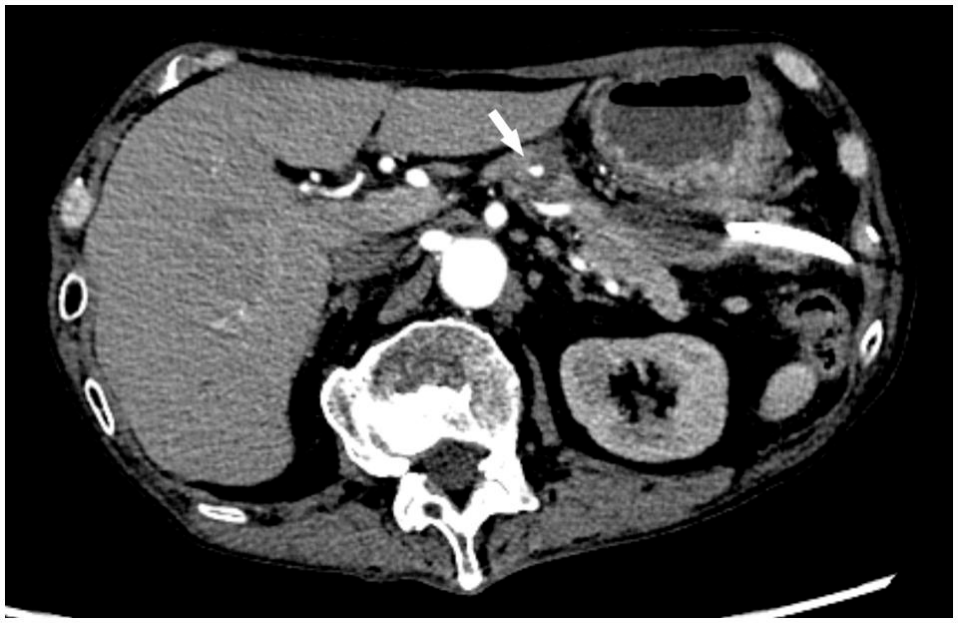
Arterial-phase, dynamic contrast-enhanced, axial computed tomography image obtained at the second admission shows a pseudoaneurysm (arrow) in the pancreatic cyst.

Three months after the discharge, the patient experienced recurring abdominal pain and was admitted to the emergency department. Blood tests revealed a lowered haemoglobin level (5.5 g/dL) and elevated serum pancreatic enzyme levels (amylase: 640 U/L, lipase: 884 U/L). Dynamic CT revealed a haematoma within the lesser sac and a pancreatic cyst, seemingly interconnected (Figure 3). Because no active bleeding was observed, a percutaneous haematoma drainage was performed. The drainage fluid was rich in pancreatic enzymes, suggesting that the pancreas was the source of the haemorrhage. Three weeks after the second admission, the sudden abdominal pain recurred. Subsequent dynamic CT revealed a pseudoaneurysm in the pancreatic cyst (Figure 4), prompting angiography.

**Figure 5.**
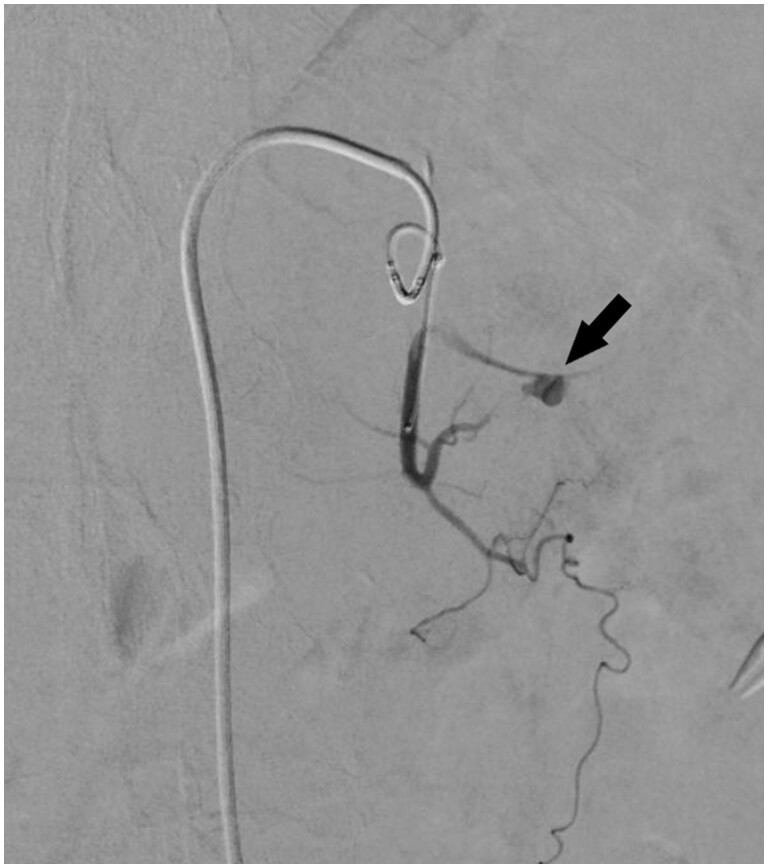
Digital subtraction angiography of the dorsal pancreatic artery shows a pseudoaneurysm (arrow) originating from the transverse pancreatic artery.

The pseudoaneurysm was located in the transverse pancreatic artery (TPA) originating from the dorsal pancreatic artery (DPA; Figure 5). Owing to the difficulties in selecting the TPA using a microcatheter, the pseudoaneurysm was embolized from the DPA using a gelatine sponge. After the second TAE, the patient recovered without any complications. No signs of recurrence were observed during the regular outpatient follow-up for 1 year.

## Discussion

hepatocellular carcinomas have a typical enhancement pattern; this enables differentiation of malignant tissue from normal liver parenchyma. Detection of focal lesions and haemorrhage is key to diagnosing an HCC rupture.^4^ Iwasaki et al reported CT manifestations in 5 cases of an HCC rupture in the caudate lobe of the liver.^5^ All cases showed exophytic tumours in the caudate lobe and high-attenuation haematomas in the lesser sac. Anatomically, the space around the caudate lobe of the liver is connected to the usually collapsed lesser sac.^6^ In our case, no focal lesions were observed in the liver, and the perihepatic haematoma compressed the liver parenchyma. Therefore, the following 2 considerations could be made retrospectively: (1) the haematoma originated from the same source as the lesser-sac haematoma and (2) the abnormal caudate lobe enhancement during angiography was simply representative of liver parenchyma congestion. Jeffrey et al reviewed 14 patients with 15 surgically proven lesions of the lesser sac.^7^ They reported that acute pancreatitis and its complications were the most common underlying causes of lesser-sac lesions. Therefore, the pancreas should have been considered an alternative source of the haematoma around the caudate lobe of the liver.

In the present case, the link between the caudate lobe haematoma and the pancreatic cyst was visualized during the initial CT examination (Figure 1). This was an important finding because pseudoaneurysms usually occur in proximity of pseudocysts. In a literature review of pseudoaneurysms of the splenic artery by Tessier et al, 64 out of 157 patients with pseudoaneurysms had concomitant pseudocysts.^8^ In the present case, it was difficult to initially conclude that the tiny, nonspecific cyst was a pseudocyst. However, it was possible to deem the cyst a pseudocyst related to pancreatitis retrospectively, because the pancreatic enzyme levels were elevated at the first admission. Conversely, a pseudoaneurysm was not apparent during the first CT examination. However, similar situations, wherein pancreatic pseudoaneurysms are not detected in case of haemorrhage from the pancreas, are sometimes observed in patients with haemosuccus pancreaticus.^9^ This observation suggests that pancreatic pseudoaneurysms may not always be detectable, possibly because of the timing of imaging evaluation. Considering the difficulties in detecting the source of the haemorrhage by imaging, abdominal paracentesis and pancreatic enzyme measurements were warranted at the first presentation.

In the present case, the pancreatic pseudoaneurysm was effectively treated with TAE alone. TAE has evolved into a first-line treatment for the management of pancreatic pseudoaneurysms owing to advances in imaging technology and interventional radiology. However, surgery remains an important treatment modality, particularly in haemodynamically compromised patients or when angiographic management fails.^10^ Had the pseudoaneurysm not been visualized at the second admission in the current case, surgical management would have been considered, because bleeding would have recurred and TAE (performed without identifying the responsible artery) would have been unsuccessful.

## Learning points

- Even if a haematoma is detected around the liver in patients with a history of alcoholic cirrhosis, causes other than an HCC rupture should be considered if lesions with enhancement patterns typical to HCCs are absent in the liver.
- The space around the caudal hepatic lobe is connected to the lesser sac.
- When a haematoma is observed around the caudal hepatic lobe, the possibility of pancreatic haemorrhage should be considered, even in the absence of detectable pseudoaneurysms or extravasation.
- TAE is useful for the management of pancreatic pseudoaneurysms; however, surgery should be considered if the patient is haemodynamically unstable or if angiographic management fails.
